# Recent Advances on the Pathobiology and Treatment of Multiple Myeloma

**DOI:** 10.3390/cancers13133112

**Published:** 2021-06-22

**Authors:** Nicola Amodio

**Affiliations:** Department of Experimental and Clinical Medicine, Magna Graecia University of Catanzaro, 88100 Catanzaro, Italy; amodio@unicz.it

Worldwide experts in the field of multiple myeloma (MM) have promptly answered to the call in the Special Issue entitled “Recent advances on the pathobiology and treatment of multiple myeloma”, submitting basic, translational or clinical works under the form of original article, review or perspective.

MM is a complex, and still incurable, hematological malignancy of terminally differentiated antibody-producing plasma cells (PCs). The reports published in this Special Issue confirm the huge effort the myeloma scientific community is presently providing towards the dissection of the bone marrow (BM) niche sustaining MM, as well as to the characterization and therapeutic targeting of the MM genomic and epigenomic landscape.

## 1. Signals Arising from the MM Bone Marrow Microenvironment (BMM)

Signals from the BMM and failure of immune surveillance are acknowledged as relevant players in MM pathogenesis and progression. In particular, the complex interaction with bone marrow stromal cells (BMSCs) strongly supports the survival, proliferation and migration of malignant PCs and promotes osteoclastogenesis and angiogenesis. Mekhloufi et al. disclosed a previously unknown immune regulatory role of BMSCs, capable of tuning MM PCs-NK cells cross-talk; in fact, IL-8 secretion by BMSCs triggered an NF-κB dependent expression of PVR ligands on MM PCs, in turn promoting DNAM-1 receptors’ recognition and NK cell degranulation [1].

“Don’t eat me” signals on MM cells, such as CD47, represent relevant factors promoting immune evasion within the BM niche. Shi et al. reported original findings on the progressive up-regulation of CD47 during MM progression, which appears amenable of pharmacological intervention using anti-CD47 blocking antibodies, rapidly inducing phagocytosis and killing of MM cells in a 3D-tissue engineered model [2].

During MM progression, the so-called angiogenic shift occurs in the BMM, characterized by the increased capability of endothelial cells (ECs) to organize a network, migrate and express angiogenic factors fostering tumor cell proliferation. Palano et al. reported that MM PCs-secreted Jagged 1 and 2 Notch ligands promoted angiogenesis in vitro as well as in a zebrafish MM model in vivo, thus representing novel targets to antagonize BM angiogenesis [3]. In parallel, Rao et al. identified the HB-EGF-EGFR signaling pathway as relevant for MM angiogenesis, as shown by high levels of EGFR and HB-EGF in BM ECs from MM compared with monoclonal gammopathy of undetermined significance patients. Blockade of HB-EGF–EGFR signaling, either by an anti-HB-EGF neutralizing antibody or the EGFR inhibitor erlotinib, dampened BM ECs angiogenic potential and hampered tumor growth, encouraging the clinical investigation of EGFR inhibitors for MM therapy [4].

The harmonious coupling of osteoclast (OC) and osteoblast (OB) activities, which regulates bone remodeling, is lost in the BMM of patients with MM bone disease (MMBD). Increased OC formation and suppressed OB activity leads to increased bone resorption that is not compensated for by bone formation.

Increasing knowledge of MMBD pathophysiology have improved the diagnostic modalities and therapeutic options. Rasch et al. review the major clinical achievements in the understanding, diagnosis and treatment of MMBD, supporting low-dose whole-body CT over conventional skeletal survey for MMBD diagnosis, along with MRI and PET/CT, that are also emerging for clinical assessment and monitoring. Bisphosphonates have for long time played a key role in the therapeutic management of MMBD, but denosumab is an alternative especially in patients with renal disease [5].

Molecular and cellular mechanisms underlying MMBD are under deep investigation and have disclosed novel therapeutic targets. Receptor activator of NF-κB ligand (RANKL), a critical mediator of osteoclastogenesis, is up-regulated in MM. Ashtar et al. reported that febuxostat, a xanthine oxidase inhibitor used for prevention of tumor lysis syndrome, antagonized RANKL-dependent osteoclastogenesis through inhibition of RANKL-induced ROS production and OC formation in vitro and in ovariectomized mice. Interestingly, doxorubicin further enhanced RANKL-induced osteoclastogenesis through up-regulation of ROS production, which was abolished by febuxostat [6]. Tibullo et al. also focused on MMBD, demonstrating that ixazomib, a third-generation proteasome inhibitor (PI), was able to reduce differentiation of human monocytes into OCs and to inhibit the expression of OC markers in vitro; moreover, ixazomib was able to stimulate osteogenic differentiation of human mesenchymal stromal cells (MSCs), increasing osteogenic markers. The authors demonstrated that PCs regulate Sonic Hedgehog signaling in MSCs acting as GLI1 suppressors, reducing the potential of MSCs to differentiate into OBs; conversely, ixazomib was able to bind the Smoothened (SMO) receptor leading to nuclear translocation of GLI1 in human MSCs, fostering osteoblastogenesis [7].

Extracellular vesicles (EVs) have been identified in the BMM and recently emerged as inducers of MMBD by inhibiting the osteogenic differentiation of human mesenchymal stem cells (hMSCs). Raimondo et al. characterized EVs released by smoldering MM (SMM) and MM cells, and identified a pool of miRNAs as EV cargo. They characterized miR-129-5p as enriched in MM-EVs compared to SMM-EVs, and demonstrated its transfer into hMSCs, where it inhibited the expression of the transcription factor Sp1, a positive modulator of OB differentiation, and of its target Alkaline phosphatase (ALPL) [8]. By proteomics, Raimondi et al. characterized the EV protein cargo, identifying UPR signaling molecules. MM-EVs administration in a murine macrophage cell line rapidly induced activation of IRE1α, along with Xbp1 mRNA splicing and transcription of NFATc1, a master transcription factor triggering OC differentiation. Importantly, GSK2850163, a chemical inhibitor of IRE1α, antagonized MM EV-triggered OC differentiation, hampering the terminal stages of OC differentiation and reducing bone resorption, strengthening Xbp1/IRE1 axis as novel OC promoter [9].

Finally, Capp et al. provided an intriguing bone-based theory for myelomagenesis, called tissue disruption-induced cell stochasticity (TiDiS), considering bone tissue as causal of the disease because of its ability to model MM oncogenesis by reconciling bone changes with genetics of MM PCs. According to TiDiS, MM should be considered a tissue-dependent process in which a critical initiating/promoting role is played by the disruption of an environmental niche. Such theory starts with the observation that the BM endosteal niche controls differentiation; as decrease in cellular stochasticity occurs thanks to cellular interactions among differentiating cells, memory B cells and plasmablasts would compete for localizing in endosteal niches, with the risk that some cells cannot fully differentiate if cannot properly seed in the niche because of a disrupted microenvironment. Therefore, such cells would remain in an unstable state with residual proliferation, with the risk that subclones may emerge and transform into cancer cells [10]. Indeed, biological and molecular correlates of TiDiS are required to validate such intriguing theory.

## 2. Therapeutic Targeting of the MM Aberrant Genomic/Epigenomic Landscape

Ninkovic & Qash reviewed the multifaceted landscape of MM, discussing the most common genomic aberrations, such as hyperdiploidy, chromosomal translocations, copy number variations, somatic mutations, as well as the contribution of the immune and non-immune tumor microenvironment [11]. All these features overall impact on the therapy of MM patients, which differs between transplant eligible (TE) and non-eligible patients. Collectively, MM patients have largely benefitted from the introduction of novel agents, basically immunotherapeutics and small molecules that have provided unprecedented outcomes.

However, the approval of several new drugs, along with the limited availability of clinical trials comparing various head-to-head possible combinations, make the therapeutic choice at each stage of the disease really complex, as underlined by Bobin et al. in their review [12]. Legarda et al. instead provide an additional thorough description of the novelties regarding therapeutic modalities against MM, remarking interesting results in TE-patients obtained with the new quadruplet therapies including monoclonal antibodies, D-VTd and D-VRd [13].

PIs have become a milestone for the treatment of multiple myeloma (MM) [14], although can induce adverse events that potentially lead to early discontinuation of the therapy with a negative impact on the quality of life and patients’ outcome, including peripheral neuropathy, cardiovascular and muscular toxicities. Pancheri et al. discuss all these side effects, focusing on the underlying cellular and molecular mechanisms which point to mitochondria dysfunction as major player [15]. Astarita et al. focused on the cardiovascular effects of the PI carfilzomib, and carried out a prospective study to assess the effectiveness of the European Myeloma Network protocol (EMN). Importantly, this study underscored the usefulness of EMN to estimate the baseline risk of cardiovascular adverse events during carfilzomib therapy, allowing the identification of higher-risk patients [16].

Regarding elderly patients, the choice of treatment goals and intensity remains challenging, and requires a multidimensional evaluation. Bonello et al. discuss the recently introduced “International Myeloma Working Group frailty score”, which identifies intermediate-fit and frail patients requiring gentler treatment approaches compared to fit patients, aiming to preserve quality of life and prevent toxicities [17]. Unfortunately, most of the anti-MM therapeutics lack effective and selective plasma cell targeting, have low therapeutic index and show poor solubility in water-based solvents. To overcome these drawbacks, Iannazzo et al. highlighted the potential usefulness of liposomes, micelles, polymeric nanoparticles, inorganic nanoparticles and carbon-based nanomaterials that have been successfully tested in MM preclinical models to improve targeting of tumor PCs [18]. Furthermore, interesting original data are presented by Nigro et al., who reported that bortezomib-loaded mesoporous silica nanoparticles, grafted with folic acid, selectively target MM cells, altering cell metabolism and triggering cell death, while sparing folate receptor negative non-tumor cells [19].

Drugs targeting aberrantly expressed, or functionally deregulated, epigenetic molecules are widely used in MM. Caprio et al. provided an overview of the most relevant epigenetic aberrations underlying MM onset and progression, along with the therapeutic approaches targeting epi-regulators. Emphasis has also been given to a class of short non-coding RNAs, named epi-microRNAs, found aberrantly expressed in MM and modifying the whole MM epigenome through the targeting of DNA methyltransferases (DNMTs) and histone deacetylases (HDACs) [20]. In this regard, Morelli et al. provided a comprehensive description of the biological and clinical impact of the dysregulated non-coding RNome in PC dyscrasias, discussing the preclinical research on all the classes of short and long non-coding RNAs (lncRNA), and analyzing the novel therapeutic approaches, mainly based on ASOs and small molecules, targeting oncogenic ncRNAs [21]. Within the same area of investigation, Ronchetti et al. unraveled the contribution of oncogenic lncRNAs to MM pathobiology, by analyzing the biological impact of ST3 beta-galactoside alpha-2,3 sialyltransferase 6 antisense RNA 1 (ST3GAL6-AS1), abundantly expressed in MM PCs; importantly, ST3GAL6-AS1 silencing via selective LNA gapmeRs dampens signaling pathways relevant for MM cell survival and proliferation [22]. In the study by Jakobsen et al., the authors instead focused on the still neglected family of circRNAs, and identified genome-wide circRNA expression patterns associated with immunomodulatory (Imid) drugs (lenalidomide and pomalidomide) responsiveness; ciRS-7 (also known as CDR1as) was highlighted as the most significantly down-regulated circRNA upon acquired Imid resistance, although its precise role in MM pathobiology requires further mechanistic investigation [23].
